# Investigation of gene–environment interactions in relation to tic severity

**DOI:** 10.1007/s00702-021-02396-y

**Published:** 2021-08-13

**Authors:** Mohamed Abdulkadir, Dongmei Yu, Lisa Osiecki, Robert A. King, Thomas V. Fernandez, Lawrence W. Brown, Keun-Ah Cheon, Barbara J. Coffey, Blanca Garcia-Delgar, Donald L. Gilbert, Dorothy E. Grice, Julie Hagstrøm, Tammy Hedderly, Isobel Heyman, Hyun Ju Hong, Chaim Huyser, Laura Ibanez-Gomez, Young Key Kim, Young-Shin Kim, Yun-Joo Koh, Sodahm Kook, Samuel Kuperman, Bennett Leventhal, Marcos Madruga-Garrido, Athanasios Maras, Pablo Mir, Astrid Morer, Alexander Münchau, Kerstin J. Plessen, Veit Roessner, Eun-Young Shin, Dong-Ho Song, Jungeun Song, Frank Visscher, Samuel H. Zinner, Carol A. Mathews, Jeremiah M. Scharf, Jay A. Tischfield, Gary A. Heiman, Andrea Dietrich, Pieter J. Hoekstra

**Affiliations:** 1 Department of Child and Adolescent Psychiatry, University Medical Center Groningen, Hanzeplein 1, 9713 GZ Groningen, The Netherlands; 2 Department of Genetics and the Human Genetics Institute of New Jersey, Rutgers, The State University of New Jersey, Piscataway, NJ 08854, USA; 3 Department of Neurology, Center for Genomic Medicine, Massachusetts General Hospital, Harvard Medical School, Boston, MA 02114, USA; 4 Department of Psychiatry, Psychiatric and Neurodevelopmental Genetics Unit, Massachusetts General Hospital, Harvard Medical School, Boston, MA 02114, USA; 5 Yale Child Study Center, Yale University School of Medicine, New Haven, CT 06510, USA; 6 Pediatric Neuropsychiatry Program, Division of Neurology, The Children’s Hospital of Philadelphia, Philadelphia, PA 19104, USA; 7 Yonsei University College of Medicine, Severance Hospital, Seoul 120-752, South Korea; 8 Icahn School of Medicine at Mount Sinai, New York, NY, USA; 9 Nathan S. Kline Institute for Psychiatric Research, Orangeburg, NY, USA; 10 University of Miami Miller School of Medicine, Miami, FL 33146, USA; 11 Department of Child and Adolescent Psychiatry and Psychology, Institute of Neurosciences, Hospital Clinic Universitari, Barcelona, Spain; 12 Cincinnati Children’s Hospital Medical Center, Cincinnati, OH, USA; 13 Child and Adolescent Mental Health Center, Mental Health Services, Capital Region of Denmark and Faculty of Health Sciences, University of Copenhagen, Copenhagen, Denmark; 14 Evelina London Children’s Hospital GSTT, Kings Health Partners AHSC, London, UK; 15 Psychological Medicine, UCL Great Ormond Street Institute of Child Health, London, UK; 16 Department of Psychiatry, Hallym University Sacred Heart Hospital, Anyang, Gyeonggi 14068, South Korea; 17 Academic Center for Child and Adolescent Psychiatry De Bascule, 1105 AZ Amsterdam, The Netherlands; 18 Family Health Centers at NYU Langone, Brooklyn, NY 11220, USA; 19 Department of Psychiatry, Yonsei Bom Clinic, Seoul 03330, South Korea; 20 University of California San Francisco Medical Center, San Francisco, CA 94143, USA; 21 Korea Institute for Children’s Social Development, Seoul, South Korea; 22 Yonsei-Nuri Mental Health Clinic, Seoul 08005, South Korea; 23 Department of Psychiatry, Carver College of Medicine, University of Iowa, Iowa, IA 52242, USA; 24 Sección de Neuropediatría, Instituto de Biomedicina de Sevilla, Hospital Universitario Virgen del Rocío/CSIC/Universidad de Sevilla, Seville, Spain; 25 Yulius Academy, Yulius Mental Health Organization, 3311 JG Dordrecht, The Netherlands; 26 Unidad de Trastornos del MovimientoInstituto de Biomedicina de Sevilla (IBiS). Hospital Universitario Virgen del Rocío/CSICUniversidad de Sevilla, Seville, Spain; 27 Department of Child and Adolescent Psychiatry and Psychology, Institute of Neurosciences, Hospital Clinic Universitari Barcelona, Spain; Institut d’Investigacions Biomediques August Pi i Sunyer (IDIPABS) and Centro de Investigacion en Red de Salud Mental (CIBERSAM), Barcelona, Spain; 28 Institute of Systems of Motor Science, University of Lübeck, 23562 Lubeck, Germany; 29 Department of Child and Adolescent Psychiatry, TU Dresden, Dresden, Germany; 30 Yonsei University Severance Hospital, Seoul 03722, South Korea; 31 Department of Psychiatry, National Health Insurance Service Ilsan Hospital, Goyang, Gyeonggi 10444, South Korea; 32 Admiraal De Ruyter Ziekenhuis, Department of Neurology, Goes, The Netherlands; 33 Department of Pediatrics, Division of Developmental Medicine, University of Washington School of Medicine, 1925 NE Pacific Street, Box 356524, Seattle, WA 98195, USA; 34 Department of Psychiatry, Center for OCD, Anxiety and Related Disorders, and Genetics Institute, University of Florida College of Medicine, Gainesville, FL 32611, USA

**Keywords:** Gene–environment interaction, Pre- and perinatal complications, Tic severity, Tourette syndrome

## Abstract

Tourette syndrome (TS) is a neuropsychiatric disorder with involvement of genetic and environmental factors. We investigated genetic loci previously implicated in Tourette syndrome and associated disorders in interaction with pre- and perinatal adversity in relation to tic severity using a case-only (*N* = 518) design. We assessed 98 single-nucleotide polymorphisms (SNPs) selected from (I) top SNPs from genome-wide association studies (GWASs) of TS; (II) top SNPs from GWASs of obsessive–compulsive disorder (OCD), attention-deficit/hyperactivity disorder (ADHD), and autism spectrum disorder (ASD); (III) SNPs previously implicated in candidate-gene studies of TS; (IV) SNPs previously implicated in OCD or ASD; and (V) tagging SNPs in neurotransmitter-related candidate genes. Linear regression models were used to examine the main effects of the SNPs on tic severity, and the interaction effect of these SNPs with a cumulative pre- and perinatal adversity score. Replication was sought for SNPs that met the threshold of significance (after correcting for multiple testing) in a replication sample (*N* = 678). One SNP (rs7123010), previously implicated in a TS meta-analysis, was significantly related to higher tic severity. We found a gene–environment interaction for rs6539267, another top TS GWAS SNP. These findings were not independently replicated. Our study highlights the future potential of TS GWAS top hits in gene–environment studies.

## Introduction

Tourette syndrome (TS) is a childhood onset neuropsychiatric disorder influenced by both genetic and environmental factors (Robertson et al. 2017). There is clear evidence that implicates both common and rare variants in TS (Qi et al. 2017; Yu et al. 2019); however, specific genetic variants only account for a small proportion of total TS disease risk. We investigated the involvement of common SNPs in candidate genes previously implicated in TS and top SNPs from GWAS of TS and comorbid disorders, and found no convincing support for these common variants (Abdulkadir et al. 2018). However, we cannot rule out that these common SNPs might yet confer risk for TS through interaction with environmental factors. Currently, gene–environment (GxE) studies are lacking and only a few small-sampled studies have investigated the genetic etiology of tic severity, suggesting involvement of the dopamine transporter gene (Tarnok et al. 2007) and the dopamine receptor D2 gene (Comings et al. 1991). Unfortunately, no GxE studies have attempted to replicate these initial findings (Qi et al. 2017). Environmental risk factors such as pre- and perinatal risk factors are also implicated in TS (Mathews et al. 2014); two studies suggested a role for a cumulative score of adverse pre- and perinatal events in TS (Abdulkadir et al. 2016; Brander et al. 2018).

The aim of the present study was to investigate whether previously implicated SNPs from genome-wide association studies and candidate-gene studies, alone and in interaction with a cumulative pre- and perinatal adversity score, are associated with lifetime tic severity using TS cases recruited by the Tourette International Collaborative Genetics (TIC Genetics) study (Dietrich et al. 2015).

## Methods

### Study subjects

This study included 586 cases (66.7% male; mean age 23.6 years, SD = 16.7, range 3–79 years) affected with a chronic tic disorder (458 with TS and 128 with chronic motor or vocal tic disorder) from the ongoing TIC Genetics study (Dietrich et al. 2015). As a replication sample, subjects were utilized from the first published TS GWAS (Scharf et al. 2013), including 678 cases (77% male; mean age 18.8 years, SD = 14, range 4–78 years) diagnosed with TS (Scharf et al. 2013).

All adult participants and parents of children provided written informed consent along with written or oral assent of their participating child. The Institutional Review Board of each participating site had approved the study.

### Diagnostic assessment

Lifetime worst-ever tic severity (mean 15.6; SD = 8.22, range 0–30) was assessed based on a modified version of the Yale Global Tic Severity Scale (Dietrich et al. 2015). The replication sample included additional items (i.e., number of tics, complexity of tics, and impairment). The mean of both parents’ education level was used as a proxy for socioeconomic status (SES).

### Cumulative pre- and perinatal adversity score

A cumulative pre- and perinatal adversity score (mean 3.52; SD = 3.42, observed range 0–21; previously described in (Abdulkadir et al. 2016)) was constructed from addition of 38 possible adverse events as measured by the self-report or parent-on-child report version of the Modified Schedule for Risk and Protective Factors Early in Development questionnaire (Walkup and Leckman 1988). Missing values were categorized as absent (coded as 0). The replication sample (Scharf et al. 2013) used the same questionnaire (Walkup and Leckman 1988) in constructing the cumulative perinatal adversity score.

### Selection of single-nucleotide polymorphisms

Genetic variants were selected based on a literature review and described in detail elsewhere (Abdulkadir et al. 2018). Briefly, a total of 196 SNPs were assessed: 12 top SNPs from the prior TS GWAS (Scharf et al. 2013; Paschou et al. 2014); 17 top SNPs from GWAS of obsessive–compulsive disorder (OCD; Stewart et al. 2013), attention-deficit/hyperactivity disorder (ADHD; Mick et al. 2011; Hinney et al. 2011), and autism spectrum disorder (ASD; Wang et al. 2009; Anney et al. 2012); 17 SNPs from candidate genes previously implicated (*P* < 0.05; Abdulkadir et al. 2018) in TS; 2 individual candidate SNPs implicated in OCD and one in ASD (Abdulkadir et al. 2018); and 148 tagging SNPs covering seven neurotransmitter-related candidate genes that were either associated with TS, OCD, or ASD (Abdulkadir et al. 2018).

### Genotyping and quality control

Genotyping of 192 SNPs (Table S1) was performed on the Illumina GoldenGate Genotyping Assay for a subset of the cases (*N* = 464). Our sample was enriched by *N* = 122 cases genotyped on the HumanOmniExpressExome v1.2 BeadChip genotyping array for a subset of the SNPs (*N*_SNPs_ = 75) available on the Goldengate Assay and four SNPs that were not present on the Goldengate Assay. The total number of SNPs genotyped across both platforms was 196. Standard quality control checks were performed with PLINK (described in detail by Abdulkadir et al. 2018), which resulted in removal of 10 SNPs. We also removed SNPs with a genotype count less than 20 (*N* = 80 SNPs) and SNPs located on the X chromosome (*N* = 8 SNPs) reducing the number of SNPs to 98 (Table S2).

### Statistical analyses

We conducted case-only analyses of tic severity using linear regressions in R (corrected for age, sex, and SES) examining; (I) the main effects of the SNPs on tic severity; and (II) the interaction effect of these SNPs with a cumulative pre- and perinatal adversity score. SNPs were coded as 0 = major allele homozygous (the reference category), 1 = heterozygous, and 2 = minor allele homozygous. Potential confounding due to relatedness of several cases was examined using mixed model analyses with familial relatedness as a random effect.

SNPs were selected from five a priori defined groups (Table S2) and we therefore applied correction for multiple testing, first, at the group level by dividing *P* = 0.05 by the number of SNPs contained within each category; referred to as *P*_group_ corrected. To correct for the number of groups tested, we further divided the obtained *P*_group_ corrected by the number of groups (i.e., five) tested; referred to as the *P*_all_. These groups were (I) top SNPs from GWAS of TS, *P*_group_ corrected = 0.0071, *P*_all_ = 0.0014 (II) top SNPs from GWAS of OCD, ADHD, and ASD, *P*_group_ corrected = 0.0063, *P*_all_ = 0.0013; (III) SNPs previously implicated in candidate-gene studies of TS *P*_group_ corrected = 0.005, *P*_all_ = 0.001, (IV) SNPs previously implicated in OCD, or ASD, *P*_group_ corrected = 0.0167, *P*_all_ = 0.0033; and (V) tagging SNPs in neurotransmitter-related candidate genes, *P*_group_ corrected = 0.0007, *P*_all_ = 0.0001. For SNPs that met the threshold of multiple testing, replication was sought in an independent sample (Scharf et al. 2013).

## Results

### Sample description

Cases missing clinical or demographic information (*N* = 68) were excluded, leaving 518 cases eligible for analyses. Results from the mixed model analyses in which a random intercept was included for familial relatedness gave similar results to the models without the random effect (Table S3, S4).

### Main effect SNPs

We found a significant association between rs7123010, a top SNP from a GWAS of TS, and tic severity, also after correction for multiple testing (*F* = 7.99, *P* = 0.0004; Tables 1, 2, and Table S1); the AA genotype was positively associated with tic severity. Results did not differ when we corrected for multiple comparisons using the less stringent Benjamini–Hochberg False Discover Rate.

### Gene-environment interaction

**Fig. 1:**
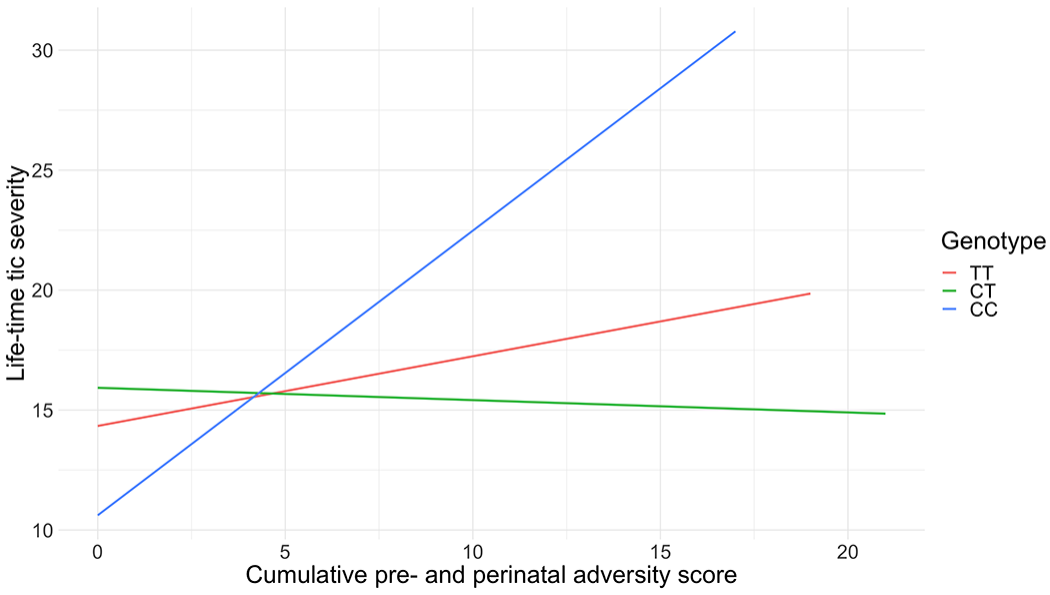
Interaction analyses of rs6539267 with a cumulative pre- and perinatal adversity score in relation to lifetime tic severity

We found a significant interaction of rs6539267, a top SNP from a TS GWAS (*F* = 6.80, *P* = 0.001) with the cumulative pre- and perinatal adversity score, also after correction for multiple testing (Tables 3, 4; Fig. 1); the CC genotype along with a higher number of pre- and perinatal adversities was positively associated with tic severity (Table 4). We found no significant interaction for rs7123010 (*F* = 0.0197, *P* = 0.98). For the GxE analysis, the pattern of results remained when we corrected for multiple comparisons using the less stringent Benjamini–Hochberg False Discover Rate.

### Replication rs7123010 and rs6539267

Investigating the main effect of rs7123010 and the interaction between rs6539267 and the cumulative pre- and perinatal adversity score in the replication sample (Scharf et al. 2013) did not show a statistically significant association (*F* = 1.98, *P* = 0.14) and (*F* = 1.29, *P* = 0.28), respectively (Table 2).

## Discussion

We investigated whether previously implicated SNPs (i) are associated with lifetime worst-ever tic severity and (ii) might interact with a cumulative pre- and perinatal adversity score previously reported to be associated with TS (Abdulkadir et al. 2016). We report a significant main effect of rs7123010 (a top TS GWAS SNP). We found no evidence for an interaction between rs7123010 and pre- and perinatal adversity. However, we did find a significant interaction between rs6539267 (another top TS GWAS SNP) and pre- and perinatal adversity. We could not confirm these findings in our replication sample (Scharf et al. 2013).

The SNP rs7123010 is located within the *ME3* (Malic Enzyme 3) gene which encodes for a protein responsible for malate metabolism (Hsieh et al. 2019). The *ME3* gene is reported to be expressed in several tissues including the brain, and the protein encoded by this gene is thought to be involved in several biological processes including fatty acid biosynthesis and insulin secretion (Hasan et al. 2015; Hsieh et al. 2019). However, there is no evidence in the literature supporting a role of *ME3* in TS. The other significant SNP in this study, rs6539267, is located within the *POLR3B* gene that encodes for the second-largest catalytic subunit of RNA polymerase III, an enzyme involved in transcription of noncoding RNAs including transfer RNAs, small ribosomal RNAs, and microRNAs (Tétreault et al. 2011; Djordjevic et al. 2021). Mutations in *POLR3B* are reported to cause hypomyelinating leukodystrophy type 8 and the clinical presentations of these mutations are widespread and include ataxia, spasticity, variable intellectual disability and epilepsy, and demyelinating sensory motor peripheral neuropathy (Djordjevic et al. 2021). Despite the wide range of the clinical manifestations of *POLR3B* mutations, tics are not considered one of them.

A plausible explanation for the non-significant replication of rs7123010 and rs6539267 is that tic severity is likely a polygenic trait and that single SNPs only account for a small fraction of the total trait variance. Furthermore, statistical power could have also been an issue; the number of individuals with a homozygous genotype of the effect alleles was quiet low for SNPs rs7123010 (the AA genotype was present in about 10% of the individuals in the initial sample and the replication sample) and rs6539267 (the CC genotype was present in about 8% of the individuals in the initial sample and in 11% of the individuals in the replication sample).

This study benefitted from use of a well-characterized sample, and from the case-only design that has shown to have more power to detect gene–environment interactions than a case–control study (Kraft et al. 2007). Furthermore, using tic severity might have allowed the detection of small effects of SNPs that would have been otherwise missed when investigating caseness; e.g., a significant association for the Dopamine Transporter 1 3’ variable number of tandem repeats has been found in relation to tic severity, but not in relation to the presence of TS (Tarnok et al. 2007).

Limitations of this study include the retrospective collection of lifetime tic severity and pre- and perinatal data, although evidence supports accurate maternal long-term recall of the latter (Rice et al. 2007). Measurement of lifetime tic severity differed across the study and replication samples, yet is not expected to explain current results. Finally, we cannot exclude that the investigated SNPs might interact with other environmental risk factors, such as life stress or infections.

In conclusion, the findings of this study suggest an association between rs7123010 and tic severity and potential gene–environment interactions of TS GWAS SNP rs6539267 with a cumulative pre- and perinatal adversity score in relation to tic severity. Our study highlights the future potential of common genetic risk variants in gene–environment studies in TS, perhaps through large-scale studies utilizing polygenic scores.

## Supplementary Material

Supplementary

## Figures and Tables

**Table 1:** Significant results from the main-effect analyses of previously implicated SNPs in relation to lifetime tic severity

SNP	Position	Chromosome	Gene	Category	Main effect in initial sample		Replication main effect	
					*F*	*P* ^a^	*F*	*P* ^a^
rs7123010	86,341,186	11	*ME3*	GWAS TS	7.99	0.0004*	1.98	0.14

*SNP* single-nucleotide polymorphism, *GWAS* genome-wide association study, *TS* Tourette syndrome

* Significant after correcting for multiple testing (*P*_al1_ = 0.0014)

a Analyses were corrected for age, sex, and socioeconomic status

**Table 2:** Comparison of genotypes of significant results from the main-effect analyses of previously implicated SNPs in relation to lifetime tic severity

SNP	Genotype	*N*	Initial sample					Replication sample				
			Lifetime tic severity Mean (SD)^a^	β	Standard error	*T*	*P*^b,c^ (Genotype)	*N*	β	Standard error	*T*	*P* ^b, c^ (Genotype)
rs7123010	GG	129	17.5 (7.75)					373				
	AG	131	15.5 (7.77)	− 1.76	0.87	− 2.02	0.045	341	0.47	0.61	0.78	0.44
	AA	26	21.6 (7.11)	3.92	1.49	2.62	0.009	64	− 1.10	1.10	− 1.01	0.31

*SNP* single-nucleotide polymorphism

a Lifetime worst-ever tic severity was assessed based on a modified version of the Yale Global Tic Severity Scale (Dietrich et al. 2015)

b Analyses were corrected for age, sex, and socioeconomic status

c Major allele homozygous genotype was used as the reference genotype

**Table 3:** Significant result from the interaction analyses of previously implicated SNPs with a cumulative pre- and perinatal adversity score in relation to lifetime tic severity

SNP	Position	Chromosome	Gene	Category	Initial sample		Replication sample	
					*F*	*P* ^a^	*F*	*P* ^a^
							
rs6539267	106,785,554	12	*POLR3B*	GWAS TS	6.8	0.001*	0.43	0.65

*SNP* single-nucleotide polymorphism, *GWAS* genome-wide association study, *TS* Tourette syndrome

* Significant after correcting for multiple testing (*P*_all_ = 0.0014)

a Analyses were corrected for age, sex, and socioeconomic status

**Table 4:** Comparison of genotypes of significant result from the interaction analyses of previously implicated SNPs with a cumulative pre- and perinatal adversity score in relation to lifetime tic severity

SNP	Genotype	*N*	Initial sample						Replication sample				
			Lifetime tic severity Mean (SD)^a^	Cumulative pre- and perinatal adversity score Mean (SD) ^b^	β	Standard error	*T*	*P*^c, d^ (Genotype)	*N*	β	Standard error	*T*	*P*^c, d^ (Genotype)
rs6539267	TT	250	15.3 (8.20)	3.30 (3.32)	–				352				
	CT	224	15.7 (7.80)	3.77 (3.76)	0.039	0.21	− 1.87	0.06	344	0.74	0.61	1.20	0.23
	CC	40	15.2 (9.62)	3.9 (3.75)	1.12	0.42	2.62	0.009	82	− 0.03	1.00	− 0.03	0.98

*SNP* single-nucleotide polymorphism

a Lifetime worst-ever tic severity was assessed based on a modified version of the Yale Global Tic Severity Scale (Dietrich et al. 2015)

b Cumulative pre- and perinatal adversity score (previously described in Abdulkadir et al. 2016) was constructed from addition of 38 possible adverse events as measured by the self-report or parent-on-child report version of the Modified Schedule for Risk and Protective Factors Early in Development questionnaire (Walkup and Leckman 1988)

c Analyses were corrected for age, sex, and socioeconomic status

d Major allele homozygous genotype was used as the reference genotype

## Data Availability

The clinical data and biomaterials (DNA, transformed cell lines, RNA) are part of a sharing repository located within the National Institute for Mental Health Center for Collaborative Genomics Research on Mental Disorders, USA, and are available to the broad scientific community: https://www.ncbi.nlm.nih.gov/projects/gap/cgi-bin/study.cgi?study_id=phs001423.v2.p2.
