# Supplementary material for: Investigation of gene–environment interactions in relation to tic severity
Source: J Neural Transm (Vienna). Author manuscript; Available in PMC 2021 Nov 1. (PMC8536549; doi:10.1007/s00702-021-02396-y)
Supplement: Supplementary [file NIHMS1750187-supplement-Supplementary.pdf]

Table S1: Selection of single nucleotide polymorphism prior to quality control

SNP	CHR	Gene	Array
rs11264126	chr01	DLGAP3	GoldenGate and OmniExpressExome
rs13063502	chr03		GoldenGate and OmniExpressExome
rs1442060	chr04	GABRA2	GoldenGate and OmniExpressExome
rs16859227	chr04	GABRA2	GoldenGate and OmniExpressExome
rs3849591	chr04	GABRA2	GoldenGate and OmniExpressExome
rs10064525	chr05	SLC6A3/DAT1	GoldenGate and OmniExpressExome
rs1042098	chr05	SLC6A3/DAT1	GoldenGate and OmniExpressExome
rs12516758	chr05	SLC6A3/DAT1	GoldenGate and OmniExpressExome
rs27048	chr05	SLC6A3/DAT1	GoldenGate and OmniExpressExome
rs40184	chr05	SLC6A3/DAT1	GoldenGate and OmniExpressExome
rs6350	chr05	SLC6A3/DAT1	GoldenGate and OmniExpressExome
rs6869645	chr05	SLC6A3/DAT1	GoldenGate and OmniExpressExome
rs7711337	chr05		GoldenGate and OmniExpressExome
rs9357271	chr06	BTBD9	GoldenGate and OmniExpressExome
rs7794745	chr07	CNTNAP2	GoldenGate and OmniExpressExome
rs7834018	chr08		GoldenGate and OmniExpressExome
rs10491734	chr09	SLC1A1	GoldenGate and OmniExpressExome
rs10758624	chr09	SLC1A1	GoldenGate and OmniExpressExome
rs10758631	chr09	SLC1A1	GoldenGate and OmniExpressExome
rs10815016	chr09	SLC1A1	GoldenGate and OmniExpressExome
rs10974619	chr09	SLC1A1	GoldenGate and OmniExpressExome
rs1471786	chr09	SLC1A1	GoldenGate and OmniExpressExome
rs16921457	chr09	SLC1A1	GoldenGate and OmniExpressExome
rs184204	chr09	SLC1A1	GoldenGate and OmniExpressExome
rs2150192	chr09	SLC1A1	GoldenGate and OmniExpressExome
rs2150195	chr09	SLC1A1	GoldenGate and OmniExpressExome
rs2183472	chr09	SLC1A1	GoldenGate and OmniExpressExome
rs4742004	chr09	SLC1A1	GoldenGate and OmniExpressExome
rs6476879	chr09	SLC1A1	GoldenGate and OmniExpressExome
rs7021409	chr09	SLC1A1	GoldenGate and OmniExpressExome
rs7022772	chr09	SLC1A1	GoldenGate and OmniExpressExome
rs7848533	chr09	SLC1A1	GoldenGate and OmniExpressExome
rs7856675	chr09	SLC1A1	GoldenGate and OmniExpressExome
rs7864496	chr09	SLC1A1	GoldenGate and OmniExpressExome
rs7868992	chr09	COL27A1	GoldenGate and OmniExpressExome
rs1079597	chr11	DRD2	GoldenGate and OmniExpressExome
rs10891556	chr11	DRD2	GoldenGate and OmniExpressExome
rs11607165	chr11	STIP1	GoldenGate and OmniExpressExome
rs12364283	chr11	DRD2	GoldenGate and OmniExpressExome
rs12422191	chr11	DRD2	GoldenGate and OmniExpressExome

rs12574471	chr11	DRD2	GoldenGate and OmniExpressExome
rs12575642	chr11	FERMT3	GoldenGate and OmniExpressExome
rs17529477	chr11	DRD2	GoldenGate and OmniExpressExome
rs4245148	chr11	DRD2	GoldenGate and OmniExpressExome
rs4648318	chr11	DRD2	GoldenGate and OmniExpressExome
rs7131056	chr11	DRD2	GoldenGate and OmniExpressExome
rs10879357	chr12	TPH2	GoldenGate and OmniExpressExome
rs11178993	chr12	TPH2	GoldenGate and OmniExpressExome
rs11178999	chr12	TPH2	GoldenGate and OmniExpressExome
rs12231341	chr12	TPH2	GoldenGate and OmniExpressExome
rs1386488	chr12	TPH2	GoldenGate and OmniExpressExome
rs1487275	chr12	TPH2	GoldenGate and OmniExpressExome
rs1872824	chr12	TPH2	GoldenGate and OmniExpressExome
rs297941	chr12		GoldenGate and OmniExpressExome
rs4565946	chr12	TPH2	GoldenGate and OmniExpressExome
rs4570625	chr12	TPH2	GoldenGate and OmniExpressExome
rs4760820	chr12	TPH2	GoldenGate and OmniExpressExome
rs6539267	chr12	POLR3B	GoldenGate and OmniExpressExome
rs7969998	chr12	TPH2	GoldenGate and OmniExpressExome
rs7336083	chr13		GoldenGate and OmniExpressExome
rs9593835	chr13	SLITRK1	GoldenGate and OmniExpressExome
rs9652236	chr13		GoldenGate and OmniExpressExome
rs3959666	chr15	HDC	GoldenGate and OmniExpressExome
rs4150167	chr16	TAF1C	GoldenGate and OmniExpressExome
rs11081062	chr18	DLGAP1	GoldenGate and OmniExpressExome
rs6131295	chr20		GoldenGate and OmniExpressExome
rs6587299	chr20	HRH3	GoldenGate and OmniExpressExome
rs944887	chr20	HRH3	GoldenGate and OmniExpressExome
rs4680	chr22	COMT	GoldenGate and OmniExpressExome
rs1181275	chrX	MAO-A	GoldenGate and OmniExpressExome
rs3027409	chrX	MAO-A	GoldenGate and OmniExpressExome
rs3027415	chrX	MAO-A	GoldenGate and OmniExpressExome
rs3813929	chrX	HTR2C	GoldenGate and OmniExpressExome
rs5906957	chrX	MAO-A	GoldenGate and OmniExpressExome
rs6609257	chrX	MAO-A	GoldenGate and OmniExpressExome
rs621942	chr11	PICALM	OmniExpressExome
rs7123010	chr11	ME3	OmniExpressExome
rs11603305	chr11		OmniExpressExome
rs2060546	chr12		OmniExpressExome
rs12141243	chr01	DLGAP3	GoldenGate
rs12037173	chr01	LRRC7	GoldenGate
rs2556378	chr02	BCL11A	GoldenGate
rs4675502	chr02	PARD3B	GoldenGate

rs4988462	chr03	POU1F1	GoldenGate
rs10013922	chr04	GABRA2	GoldenGate
rs279828	chr04	GABRA2	GoldenGate
rs1372472	chr04		GoldenGate
rs17537141	chr04	GABRA2	GoldenGate
rs16859354	chr04	GABRA2	GoldenGate
rs17537359	chr04	GABRA2	GoldenGate
rs2119183	chr04	GABRA2	GoldenGate
rs11503016	chr04	GABRA2	GoldenGate
rs4695148	chr04	GABRA2	GoldenGate
rs11503014	chr04	GABRA2	GoldenGate
rs3756007	chr04	GABRA2	GoldenGate
rs4307059	chr05		GoldenGate
rs13176113	chr05		GoldenGate
rs458860	chr05	SLC6A3	GoldenGate
rs250686	chr05	SLC6A3	GoldenGate
rs11564772	chr05	SLC6A3	GoldenGate
rs11564758	chr05	SLC6A3	GoldenGate
rs10052016	chr05	SLC6A3	GoldenGate
rs2550948	chr05		GoldenGate
rs12654851	chr05		GoldenGate
rs6347	chr05	SLC6A3	GoldenGate
rs2550956	chr05		GoldenGate
rs37022	chr05	SLC6A3	GoldenGate
rs2617605	chr05	SLC6A3	GoldenGate
rs2735917	chr05	SLC6A3	GoldenGate
rs27074	chr05		GoldenGate
rs466630	chr05	SLC6A3	GoldenGate
rs246993	chr05		GoldenGate
rs9499708	chr06		GoldenGate
rs1718101	chr07	CNTNAP2	GoldenGate
rs769111	chr07		GoldenGate
rs12342908	chr09	SLC1A1	GoldenGate
rs7022369	chr09	SLC1A1	GoldenGate
rs301979	chr09	SLC1A1	GoldenGate
rs12378107	chr09	SLC1A1	GoldenGate
rs2039290	chr09	SLC1A1	GoldenGate
rs10739064	chr09	SLC1A1	GoldenGate
rs10814998	chr09	SLC1A1	GoldenGate
rs12341219	chr09	SLC1A1	GoldenGate
rs10491735	chr09		GoldenGate
rs10739066	chr09	SLC1A1	GoldenGate
rs4742003	chr09	SLC1A1	GoldenGate

rs10491731	chr09	SLC1A1	GoldenGate
rs7858819	chr09	SLC1A1	GoldenGate
rs10974591	chr09	SLC1A1	GoldenGate
rs10814991	chr09	SLC1A1	GoldenGate
rs12553697	chr09		GoldenGate
rs6476873	chr09	SLC1A1	GoldenGate
rs7031998	chr09	SLC1A1	GoldenGate
rs10739062	chr09	SLC1A1	GoldenGate
rs928209	chr09	SLC1A1	GoldenGate
rs301443	chr09		GoldenGate
rs10974616	chr09	SLC1A1	GoldenGate
rs9775228	chr09		GoldenGate
rs10758632	chr09	SLC1A1	GoldenGate
rs10814993	chr09	SLC1A1	GoldenGate
rs301434	chr09	SLC1A1	GoldenGate
rs301439	chr09		GoldenGate
rs17812372	chr09		GoldenGate
rs972519	chr09	SLC1A1	GoldenGate
rs10814988	chr09		GoldenGate
rs10974620	chr09	SLC1A1	GoldenGate
rs301445	chr09		GoldenGate
rs10815013	chr09	SLC1A1	GoldenGate
rs10815019	chr09	SLC1A1	GoldenGate
rs2587548	chr11	DRD2	GoldenGate
rs2587550	chr11		GoldenGate
rs7122454	chr11	DRD2	GoldenGate
rs17115583	chr11	DRD2	GoldenGate
rs4245147	chr11	DRD2	GoldenGate
rs2234689	chr11		GoldenGate
rs1800497	chr11	ANKK1	GoldenGate
rs11214606	chr11	DRD2	GoldenGate
rs6279	chr11	DRD2	GoldenGate
rs5016282	chr11	GRM5	GoldenGate
rs4271390	chr11	PVRL1	GoldenGate
rs7955501	chr12	TPH2	GoldenGate
rs1386497	chr12	TPH2	GoldenGate
rs17110536	chr12	TPH2	GoldenGate
rs10879358	chr12	TPH2	GoldenGate
rs10748185	chr12	TPH2	GoldenGate
rs17722134	chr12	TPH2	GoldenGate
rs4760813	chr12		GoldenGate
rs17110747	chr12	TPH2	GoldenGate
rs11615016	chr12	TPH2	GoldenGate

rs12424836	chr12		GoldenGate
rs11149058	chr13		GoldenGate
rs9531520	chr13		GoldenGate
rs8029462	chr15	SLC27A2	GoldenGate
rs11856059	chr15		GoldenGate
rs1365503	chr15		GoldenGate
rs854157	chr15		GoldenGate
rs2853766	chr15		GoldenGate
rs854158	chr15		GoldenGate
rs854159	chr15		GoldenGate
rs2070595	chr15		GoldenGate
rs8034597	chr15		GoldenGate
rs854150	chr15		GoldenGate
rs9920021	chr15		GoldenGate
rs662669	chr17	TBCD	GoldenGate
rs3744161	chr17	TBCD	GoldenGate
rs3787430	chr20	HRH3	GoldenGate
rs1760042	chr20		GoldenGate
rs6062144	chr20		GoldenGate
rs6061458	chr20		GoldenGate
rs3787429	chr20	HRH3	GoldenGate
rs6142998	chr20		GoldenGate
rs518147	chrX	HTR2C	GoldenGate
rs5906729	chrX	MAO-A	GoldenGate
rs5905859	chrX	MAO-A	GoldenGate
rs3027399	chrX	MAO-A	GoldenGate
rs12843533	chrX	MAO-A	GoldenGate

[a]GoldenGate: Illumina GoldenGate Genotyping Assay; OmniExpressExome: Illumina HumanOmniExpressExome v1.2 BeadChip array.

Table S2: Investigated SNPs after quality control

SNP	Gene	Category	Reference
rs769111		Top SNP GWAS TS	(5)
rs7868992	*COL27A1*	Top SNP GWAS TS	(5)
rs11603305		Top SNP GWAS TS	(6)
rs621942	*PICALM*	Top SNP GWAS TS	(6)
rs7123010	*ME3*	Top SNP GWAS TS	(6)
rs6539267	*POLR3B*	Top SNP GWAS TS	(5)
rs7336083		Top SNP GWAS TS	(5)
rs4675502	*PARD3B*	Top SNP GWAS OCD/ADHD/ASD	(1)
rs4988462	*POU1F1*	Top SNP GWAS OCD/ADHD/ASD	(2)
rs13176113		Top SNP GWAS OCD/ADHD/ASD	(3)
rs4307059		Top SNP GWAS OCD/ADHD/ASD	(3)
rs7711337		Top SNP GWAS OCD/ADHD/ASD	(1)
rs9499708		Top SNP GWAS OCD/ADHD/ASD	(4)
rs11081062	*DLGAP1*	Top SNP GWAS OCD/ADHD/ASD	(4)
rs6131295		Top SNP GWAS OCD/ADHD/ASD	(4)
rs6347	*DAT1*	Previously implicated in candidate gene studies of TS	(8)
rs9357271	*BTBD9*	Previously implicated in candidate gene studies of TS	(9)
rs6279	*DRD2*	Previously implicated in candidate gene studies of TS	(11)
rs4648318	*DRD2*	Previously implicated in candidate gene studies of TS	(11)
rs4570625	*TPH2*	Previously implicated in candidate gene studies of TS	(12)
rs4565946	*TPH2*	Previously implicated in candidate gene studies of TS	(12)
rs9593835	*SLITRK1*	Previously implicated in candidate gene studies of TS	(13)
rs854150	*HDC*	Previously implicated in candidate gene studies of TS	(14)
rs662669	*TBCD*	Previously implicated in candidate gene studies of TS	(15)
rs3744161	*TBCD*	Previously implicated in candidate gene studies of TS	(15)
rs11264126	*DLGAP3*	Previously implicated in OCD/ASD	(7)
rs7794745	*CNTNAP2*	Previously implicated in OCD/ASD	(10)
rs4680	*COMT*	Previously implicated in OCD/ASD	(16)
rs16859227	*GABRA2*	TS neurotransmitter-related candidate genes	(17)
rs279828	*GABRA2*	TS neurotransmitter-related candidate genes	(17)
rs1442060	*GABRA2*	TS neurotransmitter-related candidate genes	(17)
rs16859354	*GABRA2*	TS neurotransmitter-related candidate genes	(17)
rs10013922	*GABRA2*	TS neurotransmitter-related candidate genes	(17)
rs11503014	*GABRA2*	TS neurotransmitter-related candidate genes	(17)
rs1372472	*GABRA2*	TS neurotransmitter-related candidate genes	(17)
rs1042098	*DAT1*	TS neurotransmitter-related candidate genes	(8)
rs40184	*DAT1*	TS neurotransmitter-related candidate genes	(8)
rs27048	*DAT1*	TS neurotransmitter-related candidate genes	(8)
rs11564758	*DAT1*	TS neurotransmitter-related candidate genes	(8)
rs10052016	*DAT1*	TS neurotransmitter-related candidate genes	(8)

rs2550948	*DAT1*	TS neurotransmitter-related candidate genes	(8)
rs10491734	*SLC1A1*	TS neurotransmitter-related candidate genes	(18)
rs9775228	*SLC1A1*	TS neurotransmitter-related candidate genes	(18)
rs2150192	*SLC1A1*	TS neurotransmitter-related candidate genes	(18)
rs2183472	*SLC1A1*	TS neurotransmitter-related candidate genes	(18)
rs7031998	*SLC1A1*	TS neurotransmitter-related candidate genes	(18)
rs10814991	*SLC1A1*	TS neurotransmitter-related candidate genes	(18)
rs10814993	*SLC1A1*	TS neurotransmitter-related candidate genes	(18)
rs7021409	*SLC1A1*	TS neurotransmitter-related candidate genes	(18)
rs10739062	*SLC1A1*	TS neurotransmitter-related candidate genes	(18)
rs2150195	*SLC1A1*	TS neurotransmitter-related candidate genes	(18)
rs12342908	*SLC1A1*	TS neurotransmitter-related candidate genes	(18)
rs10814998	*SLC1A1*	TS neurotransmitter-related candidate genes	(18)
rs4742003	*SLC1A1*	TS neurotransmitter-related candidate genes	(18)
rs4742004	*SLC1A1*	TS neurotransmitter-related candidate genes	(18)
rs7022369	*SLC1A1*	TS neurotransmitter-related candidate genes	(18)
rs7848533	*SLC1A1*	TS neurotransmitter-related candidate genes	(18)
rs928209	*SLC1A1*	TS neurotransmitter-related candidate genes	(18)
rs10758631	*SLC1A1*	TS neurotransmitter-related candidate genes	(18)
rs10815019	*SLC1A1*	TS neurotransmitter-related candidate genes	(18)
rs12341219	*SLC1A1*	TS neurotransmitter-related candidate genes	(18)
rs10739064	*SLC1A1*	TS neurotransmitter-related candidate genes	(18)
rs10758632	*SLC1A1*	TS neurotransmitter-related candidate genes	(18)
rs10739066	*SLC1A1*	TS neurotransmitter-related candidate genes	(18)
rs10974619	*SLC1A1*	TS neurotransmitter-related candidate genes	(18)
rs7858819	*SLC1A1*	TS neurotransmitter-related candidate genes	(18)
rs7022772	*SLC1A1*	TS neurotransmitter-related candidate genes	(18)
rs7864496	*SLC1A1*	TS neurotransmitter-related candidate genes	(18)
rs301979	*SLC1A1*	TS neurotransmitter-related candidate genes	(18)
rs6476879	*SLC1A1*	TS neurotransmitter-related candidate genes	(18)
rs12378107	*SLC1A1*	TS neurotransmitter-related candidate genes	(18)
rs301434	*SLC1A1*	TS neurotransmitter-related candidate genes	(18)
rs301439	*SLC1A1*	TS neurotransmitter-related candidate genes	(18)
rs301443	*SLC1A1*	TS neurotransmitter-related candidate genes	(18)
rs301445	*SLC1A1*	TS neurotransmitter-related candidate genes	(18)
rs2587550	*DRD2*	TS neurotransmitter-related candidate genes	(19)
rs2587548	*DRD2*	TS neurotransmitter-related candidate genes	(19)
rs17529477	*DRD2*	TS neurotransmitter-related candidate genes	(19)
rs4245147	*DRD2*	TS neurotransmitter-related candidate genes	(19)
rs7131056	*DRD2*	TS neurotransmitter-related candidate genes	(19)
rs11178999	*TPH2*	TS neurotransmitter-related candidate genes	(20)
rs10748185	*TPH2*	TS neurotransmitter-related candidate genes	(20)
rs7955501	*TPH2*	TS neurotransmitter-related candidate genes	(20)

rs17110536	*TPH2*	TS neurotransmitter-related candidate genes	(20)
rs4760820	*TPH2*	TS neurotransmitter-related candidate genes	(20)
rs1487275	*TPH2*	TS neurotransmitter-related candidate genes	(20)
rs10879357	*TPH2*	TS neurotransmitter-related candidate genes	(20)
rs10879358	*TPH2*	TS neurotransmitter-related candidate genes	(20)
rs1872824	*TPH2*	TS neurotransmitter-related candidate genes	(20)
rs2070595	*HDC*	TS neurotransmitter-related candidate genes	(21)
rs2853766	*HDC*	TS neurotransmitter-related candidate genes	(21)
rs854157	*HDC*	TS neurotransmitter-related candidate genes	(21)
rs854158	*HDC*	TS neurotransmitter-related candidate genes	(21)
rs854159	*HDC*	TS neurotransmitter-related candidate genes	(21)
rs9920021	*HDC*	TS neurotransmitter-related candidate genes	(21)
rs1365503	*HDC*	TS neurotransmitter-related candidate genes	(21)
rs6061458	*HRH3*	TS neurotransmitter-related candidate genes	(22)
rs6142998	*HRH3*	TS neurotransmitter-related candidate genes	(22)

SNP, singe nucleotide polymorphism; GWAS, genome-wide association study.

Table S3: Regression models main-effect analyses with and without relatedness as a random effect

	Relatedness as random effect		Without relatedness as random effect	
**SNP**	**χ**	***P***	**F**	***P***
rs7123010	7.95	0.0004	7.99	0.0004
rs11264126	4.46	0.0120	4.22	0.0152
rs6061458	0.43	0.6509	4.22	0.0152
rs17529477	4.55	0.0110	4.10	0.0171
rs7021409	2.92	0.0552	3.19	0.0421
rs928209	2.88	0.0574	3.00	0.0511
rs9920021	3.22	0.0411	2.82	0.0607
rs854158	2.38	0.0943	2.53	0.0813
rs301979	2.33	0.0988	2.30	0.1017
rs279828	2.20	0.1127	2.26	0.1057
rs301439	2.46	0.0864	2.21	0.1105
rs12341219	2.05	0.1299	2.20	0.1122
rs3744161	2.14	0.1188	2.11	0.1230
rs2587550	2.23	0.1087	2.04	0.1318
rs1372472	2.12	0.1215	2.02	0.1344
rs6476879	1.96	0.1424	2.00	0.1364
rs7848533	1.77	0.1707	1.97	0.1403
rs1442060	1.85	0.1582	1.73	0.1786
rs6279	1.92	0.1484	1.72	0.1808
rs7031998	1.65	0.1935	1.70	0.1832
rs11081062	1.72	0.1800	1.70	0.1840

rs4675502	1.58	0.2081	1.64	0.1961
rs6142998	0.33	0.7201	1.64	0.1961
rs7022772	1.68	0.1880	1.59	0.2045
rs4565946	1.66	0.1912	1.53	0.2179
rs16859227	1.47	0.2317	1.48	0.2292
rs11503014	1.50	0.2255	1.46	0.2332
rs7336083	1.36	0.2585	1.45	0.2364
rs11564758	1.32	0.2685	1.39	0.2512
rs4742003	1.32	0.2683	1.38	0.2532
rs4742004	1.22	0.2955	1.36	0.2567
rs10739064	1.20	0.3022	1.29	0.2757
rs2587548	1.16	0.3140	1.29	0.2773
rs7794745	1.37	0.2554	1.26	0.2852
rs7955501	1.17	0.3119	1.20	0.3012
rs10491734	1.29	0.2763	1.20	0.3023
rs10758632	1.14	0.3221	1.17	0.3103
rs7022369	1.24	0.2903	1.16	0.3131
rs12342908	1.10	0.3340	1.16	0.3137
rs10748185	1.17	0.3112	1.10	0.3339
rs13176113	1.09	0.3388	1.06	0.3472
rs1487275	1.07	0.3424	1.06	0.3477
rs10013922	0.95	0.3874	1.02	0.3618
rs2183472	1.00	0.3696	1.01	0.3641
rs17110536	1.07	0.3429	1.01	0.3646
rs4307059	1.05	0.3517	1.01	0.3651
rs10974619	0.94	0.3925	0.99	0.3724
rs9775228	1.01	0.3645	0.96	0.3847
rs27048	0.85	0.4275	0.96	0.3850
rs662669	0.96	0.3830	0.96	0.3852
rs10879357	0.90	0.4089	0.85	0.4280
rs301443	0.94	0.3899	0.78	0.4577
rs7868992	0.67	0.5139	0.76	0.4666
rs2150195	0.91	0.4040	0.75	0.4711
rs1042098	0.66	0.5152	0.70	0.4953
rs9499708	0.65	0.5217	0.69	0.4999
rs9593835	0.71	0.4944	0.68	0.5088
rs4680	0.36	0.6971	0.64	0.5269
rs4988462	0.69	0.5032	0.64	0.5269
rs854150	0.67	0.5147	0.61	0.5443
rs7858819	0.58	0.5625	0.59	0.5549
rs6539267	0.55	0.5784	0.55	0.5780
rs10052016	0.56	0.5725	0.53	0.5881
rs10814998	0.48	0.6193	0.53	0.5890

rs621942	0.52	0.5937	0.52	0.5944
rs2550948	0.52	0.5972	0.49	0.6136
rs10758631	0.36	0.6969	0.47	0.6274
rs16859354	0.42	0.6580	0.47	0.6284
rs7131056	0.48	0.6206	0.45	0.6384
rs40184	0.40	0.6688	0.44	0.6457
rs10815019	0.45	0.6362	0.43	0.6480
rs4570625	0.37	0.6929	0.41	0.6666
rs11178999	0.40	0.6694	0.40	0.6720
rs4245147	0.39	0.6780	0.34	0.7142
rs1872824	0.28	0.7565	0.33	0.7174
rs10814993	0.34	0.7129	0.32	0.7256
rs7864496	0.36	0.6953	0.29	0.7475
rs1365503	0.36	0.6958	0.29	0.7491
rs6347	0.25	0.7804	0.29	0.7498
rs301434	0.39	0.6768	0.28	0.7551
rs10739062	0.38	0.6856	0.27	0.7613
rs854159	0.19	0.8265	0.24	0.7897
rs2150192	0.28	0.7560	0.23	0.7945
rs10814991	0.29	0.7456	0.23	0.7983
rs9357271	0.25	0.7754	0.23	0.7986
rs2853766	0.26	0.7748	0.22	0.8002
rs2070595	0.24	0.7875	0.21	0.8072
rs301445	0.24	0.7831	0.21	0.8116
rs854157	0.15	0.8640	0.19	0.8283
rs10879358	0.21	0.8125	0.19	0.8305
rs7711337	0.13	0.8766	0.18	0.8329
rs10739066	0.26	0.7725	0.17	0.8415
rs4760820	0.12	0.8907	0.16	0.8562
rs769111	0.07	0.9337	0.07	0.9340
rs12378107	0.05	0.9486	0.06	0.9406
rs6131295	0.11	0.8994	0.05	0.9470
rs4648318	0.08	0.9207	0.03	0.9720
rs11603305	0.01	0.9869	0.01	0.9922

SNP, singe nucleotide polymorphism

Table S4: Regression models interaction analyses with and without relatedness as a random effect

	Relatedness as random effect		Relatedness as random effect	
**SNP**	**χ**	***P***	**F**	***P***
rs6539267	6.94	0.001	6.80	0.001
rs12342908	4.81	0.009	4.70	0.010
rs4742003	4.15	0.017	4.05	0.018
rs4648318	3.65	0.027	3.58	0.029
rs301979	3.60	0.028	3.51	0.031
rs2150195	3.29	0.038	3.23	0.040
rs4742004	3.19	0.042	3.13	0.045
rs2587550	2.69	0.069	2.63	0.074
rs279828	2.68	0.070	2.62	0.074
rs6279	2.59	0.076	2.53	0.081
rs10758632	2.48	0.085	2.42	0.090
rs7858819	2.32	0.100	2.27	0.105
rs10815019	2.04	0.131	2.00	0.137
rs854158	1.99	0.139	1.94	0.145
rs10814998	1.94	0.145	1.89	0.152
rs10814991	1.93	0.147	1.89	0.153
rs10013922	1.92	0.148	1.88	0.154
rs10814993	1.79	0.168	1.75	0.175
rs10739064	1.77	0.172	1.73	0.179
rs7864496	1.75	0.175	1.72	0.181
rs17529477	1.66	0.191	1.63	0.198
rs9775228	1.63	0.198	1.59	0.205
rs27048	1.52	0.221	1.49	0.227
rs40184	1.51	0.223	1.48	0.229
rs854159	1.50	0.224	1.47	0.231
rs2853766	1.48	0.228	1.46	0.234
rs4988462	1.47	0.231	1.44	0.239
rs4680	0.21	0.809	1.44	0.239
rs2183472	1.25	0.288	1.22	0.295
rs854157	1.23	0.294	1.20	0.302
rs11264126	1.16	0.315	1.14	0.322
rs6061458	2.54	0.080	1.14	0.322
rs10739062	1.11	0.331	1.08	0.339
rs2587548	1.07	0.345	1.04	0.353
rs12341219	1.00	0.368	0.98	0.376
rs7336083	1.00	0.370	0.98	0.377
rs6347	0.98	0.376	0.96	0.385
rs301434	0.91	0.402	0.89	0.410

rs11503014	0.90	0.408	0.88	0.416
rs7711337	0.87	0.419	0.86	0.426
rs9920021	0.87	0.418	0.85	0.426
rs10974619	0.87	0.421	0.85	0.428
rs1042098	0.85	0.426	0.84	0.433
rs928209	0.80	0.449	0.78	0.457
rs7131056	0.78	0.461	0.76	0.468
rs6476879	0.75	0.472	0.74	0.479
rs16859354	0.74	0.479	0.72	0.487
rs16859227	0.73	0.481	0.72	0.488
rs10879357	0.71	0.494	0.69	0.501
rs1442060	0.70	0.496	0.69	0.503
rs301439	0.70	0.496	0.69	0.504
rs10491734	0.69	0.503	0.67	0.510
rs7955501	0.67	0.512	0.66	0.520
rs11178999	0.66	0.517	0.65	0.523
rs301443	0.65	0.525	0.63	0.532
rs4570625	0.63	0.535	0.61	0.542
rs4565946	0.62	0.537	0.61	0.544
rs11603305	0.63	0.534	0.61	0.546
rs10739066	0.61	0.543	0.60	0.550
rs9499708	0.60	0.547	0.59	0.554
rs7021409	0.57	0.564	0.56	0.571
rs7848533	0.57	0.565	0.56	0.571
rs4245147	0.54	0.583	0.53	0.590
rs10748185	0.54	0.584	0.53	0.591
rs301445	0.53	0.592	0.51	0.599
rs10879358	0.52	0.592	0.51	0.599
rs17110536	0.50	0.610	0.48	0.616
rs10758631	0.49	0.611	0.48	0.616
rs7022772	0.46	0.634	0.45	0.639
rs4307059	0.45	0.635	0.44	0.642
rs4675502	0.44	0.647	0.43	0.653
rs6142998	0.22	0.803	0.43	0.653
rs1372472	0.38	0.686	0.37	0.692
rs12378107	0.37	0.690	0.36	0.696
rs7868992	0.35	0.704	0.34	0.708
rs9357271	0.33	0.721	0.32	0.726
rs621942	0.30	0.742	0.29	0.750
rs4760820	0.28	0.753	0.28	0.757
rs9593835	0.28	0.753	0.28	0.757
rs3744161	0.26	0.769	0.26	0.773
rs11564758	0.26	0.772	0.25	0.777

rs1487275	0.25	0.778	0.25	0.782
rs1872824	0.24	0.783	0.24	0.788
rs7794745	0.23	0.796	0.22	0.799
rs6131295	0.22	0.799	0.22	0.802
rs7022369	0.22	0.807	0.21	0.810
rs2070595	0.21	0.809	0.21	0.812
rs1365503	0.19	0.825	0.19	0.828
rs11081062	0.12	0.884	0.12	0.886
rs769111	0.11	0.899	0.10	0.901
rs10052016	0.09	0.913	0.09	0.915
rs2550948	0.07	0.930	0.07	0.932
rs7031998	0.05	0.951	0.05	0.952
rs13176113	0.04	0.956	0.04	0.957
rs662669	0.02	0.977	0.02	0.977
rs7123010	0.02	0.980	0.02	0.980
rs854150	0.01	0.986	0.01	0.986
rs2150192	0.01	0.993	0.01	0.993

SNP, singe nucleotide polymorphism
