# Supplementary material for: Investigation of gene–environment interactions in relation to tic severity
Source: J Neural Transm (Vienna). 2021 Aug 13;128(11):1757–65. doi: 10.1007/s00702-021-02396-y (PMC8536549; doi:10.1007/s00702-021-02396-y)
Supplement: Supplementary file 1 [file 702_2021_2396_MOESM1_ESM.docx]

Table S1: Selection of single nucleotide polymorphism prior to quality control

| SNP | CHR | Gene | Array |
| --- | --- | --- | --- |
| rs11264126 | chr01 | DLGAP3 | GoldenGate and OmniExpressExome |
| rs13063502 | chr03 |  | GoldenGate and OmniExpressExome |
| rs1442060 | chr04 | GABRA2 | GoldenGate and OmniExpressExome |
| rs16859227 | chr04 | GABRA2 | GoldenGate and OmniExpressExome |
| rs3849591 | chr04 | GABRA2 | GoldenGate and OmniExpressExome |
| rs10064525 | chr05 | SLC6A3/DAT1 | GoldenGate and OmniExpressExome |
| rs1042098 | chr05 | SLC6A3/DAT1 | GoldenGate and OmniExpressExome |
| rs12516758 | chr05 | SLC6A3/DAT1 | GoldenGate and OmniExpressExome |
| rs27048 | chr05 | SLC6A3/DAT1 | GoldenGate and OmniExpressExome |
| rs40184 | chr05 | SLC6A3/DAT1 | GoldenGate and OmniExpressExome |
| rs6350 | chr05 | SLC6A3/DAT1 | GoldenGate and OmniExpressExome |
| rs6869645 | chr05 | SLC6A3/DAT1 | GoldenGate and OmniExpressExome |
| rs7711337 | chr05 |  | GoldenGate and OmniExpressExome |
| rs9357271 | chr06 | BTBD9 | GoldenGate and OmniExpressExome |
| rs7794745 | chr07 | CNTNAP2 | GoldenGate and OmniExpressExome |
| rs7834018 | chr08 |  | GoldenGate and OmniExpressExome |
| rs10491734 | chr09 | SLC1A1 | GoldenGate and OmniExpressExome |
| rs10758624 | chr09 | SLC1A1 | GoldenGate and OmniExpressExome |
| rs10758631 | chr09 | SLC1A1 | GoldenGate and OmniExpressExome |
| rs10815016 | chr09 | SLC1A1 | GoldenGate and OmniExpressExome |
| rs10974619 | chr09 | SLC1A1 | GoldenGate and OmniExpressExome |
| rs1471786 | chr09 | SLC1A1 | GoldenGate and OmniExpressExome |
| rs16921457 | chr09 | SLC1A1 | GoldenGate and OmniExpressExome |
| rs184204 | chr09 | SLC1A1 | GoldenGate and OmniExpressExome |
| rs2150192 | chr09 | SLC1A1 | GoldenGate and OmniExpressExome |
| rs2150195 | chr09 | SLC1A1 | GoldenGate and OmniExpressExome |
| rs2183472 | chr09 | SLC1A1 | GoldenGate and OmniExpressExome |
| rs4742004 | chr09 | SLC1A1 | GoldenGate and OmniExpressExome |
| rs6476879 | chr09 | SLC1A1 | GoldenGate and OmniExpressExome |
| rs7021409 | chr09 | SLC1A1 | GoldenGate and OmniExpressExome |
| rs7022772 | chr09 | SLC1A1 | GoldenGate and OmniExpressExome |
| rs7848533 | chr09 | SLC1A1 | GoldenGate and OmniExpressExome |
| rs7856675 | chr09 | SLC1A1 | GoldenGate and OmniExpressExome |
| rs7864496 | chr09 | SLC1A1 | GoldenGate and OmniExpressExome |
| rs7868992 | chr09 | COL27A1 | GoldenGate and OmniExpressExome |
| rs1079597 | chr11 | DRD2 | GoldenGate and OmniExpressExome |
| rs10891556 | chr11 | DRD2 | GoldenGate and OmniExpressExome |
| rs11607165 | chr11 | STIP1 | GoldenGate and OmniExpressExome |
| rs12364283 | chr11 | DRD2 | GoldenGate and OmniExpressExome |
| rs12422191 | chr11 | DRD2 | GoldenGate and OmniExpressExome |
| rs12574471 | chr11 | DRD2 | GoldenGate and OmniExpressExome |
| rs12575642 | chr11 | FERMT3 | GoldenGate and OmniExpressExome |
| rs17529477 | chr11 | DRD2 | GoldenGate and OmniExpressExome |
| rs4245148 | chr11 | DRD2 | GoldenGate and OmniExpressExome |
| rs4648318 | chr11 | DRD2 | GoldenGate and OmniExpressExome |
| rs7131056 | chr11 | DRD2 | GoldenGate and OmniExpressExome |
| rs10879357 | chr12 | TPH2 | GoldenGate and OmniExpressExome |
| rs11178993 | chr12 | TPH2 | GoldenGate and OmniExpressExome |
| rs11178999 | chr12 | TPH2 | GoldenGate and OmniExpressExome |
| rs12231341 | chr12 | TPH2 | GoldenGate and OmniExpressExome |
| rs1386488 | chr12 | TPH2 | GoldenGate and OmniExpressExome |
| rs1487275 | chr12 | TPH2 | GoldenGate and OmniExpressExome |
| rs1872824 | chr12 | TPH2 | GoldenGate and OmniExpressExome |
| rs297941 | chr12 |  | GoldenGate and OmniExpressExome |
| rs4565946 | chr12 | TPH2 | GoldenGate and OmniExpressExome |
| rs4570625 | chr12 | TPH2 | GoldenGate and OmniExpressExome |
| rs4760820 | chr12 | TPH2 | GoldenGate and OmniExpressExome |
| rs6539267 | chr12 | POLR3B | GoldenGate and OmniExpressExome |
| rs7969998 | chr12 | TPH2 | GoldenGate and OmniExpressExome |
| rs7336083 | chr13 |  | GoldenGate and OmniExpressExome |
| rs9593835 | chr13 | SLITRK1 | GoldenGate and OmniExpressExome |
| rs9652236 | chr13 |  | GoldenGate and OmniExpressExome |
| rs3959666 | chr15 | HDC | GoldenGate and OmniExpressExome |
| rs4150167 | chr16 | TAF1C | GoldenGate and OmniExpressExome |
| rs11081062 | chr18 | DLGAP1 | GoldenGate and OmniExpressExome |
| rs6131295 | chr20 |  | GoldenGate and OmniExpressExome |
| rs6587299 | chr20 | HRH3 | GoldenGate and OmniExpressExome |
| rs944887 | chr20 | HRH3 | GoldenGate and OmniExpressExome |
| rs4680 | chr22 | COMT | GoldenGate and OmniExpressExome |
| rs1181275 | chrX | MAO-A | GoldenGate and OmniExpressExome |
| rs3027409 | chrX | MAO-A | GoldenGate and OmniExpressExome |
| rs3027415 | chrX | MAO-A | GoldenGate and OmniExpressExome |
| rs3813929 | chrX | HTR2C | GoldenGate and OmniExpressExome |
| rs5906957 | chrX | MAO-A | GoldenGate and OmniExpressExome |
| rs6609257 | chrX | MAO-A | GoldenGate and OmniExpressExome |
| rs621942 | chr11 | PICALM | OmniExpressExome |
| rs7123010 | chr11 | ME3 | OmniExpressExome |
| rs11603305 | chr11 |  | OmniExpressExome |
| rs2060546 | chr12 |  | OmniExpressExome |
| rs12141243 | chr01 | DLGAP3 | GoldenGate |
| rs12037173 | chr01 | LRRC7 | GoldenGate |
| rs2556378 | chr02 | BCL11A | GoldenGate |
| rs4675502 | chr02 | PARD3B | GoldenGate |
| rs4988462 | chr03 | POU1F1 | GoldenGate |
| rs10013922 | chr04 | GABRA2 | GoldenGate |
| rs279828 | chr04 | GABRA2 | GoldenGate |
| rs1372472 | chr04 |  | GoldenGate |
| rs17537141 | chr04 | GABRA2 | GoldenGate |
| rs16859354 | chr04 | GABRA2 | GoldenGate |
| rs17537359 | chr04 | GABRA2 | GoldenGate |
| rs2119183 | chr04 | GABRA2 | GoldenGate |
| rs11503016 | chr04 | GABRA2 | GoldenGate |
| rs4695148 | chr04 | GABRA2 | GoldenGate |
| rs11503014 | chr04 | GABRA2 | GoldenGate |
| rs3756007 | chr04 | GABRA2 | GoldenGate |
| rs4307059 | chr05 |  | GoldenGate |
| rs13176113 | chr05 |  | GoldenGate |
| rs458860 | chr05 | SLC6A3 | GoldenGate |
| rs250686 | chr05 | SLC6A3 | GoldenGate |
| rs11564772 | chr05 | SLC6A3 | GoldenGate |
| rs11564758 | chr05 | SLC6A3 | GoldenGate |
| rs10052016 | chr05 | SLC6A3 | GoldenGate |
| rs2550948 | chr05 |  | GoldenGate |
| rs12654851 | chr05 |  | GoldenGate |
| rs6347 | chr05 | SLC6A3 | GoldenGate |
| rs2550956 | chr05 |  | GoldenGate |
| rs37022 | chr05 | SLC6A3 | GoldenGate |
| rs2617605 | chr05 | SLC6A3 | GoldenGate |
| rs2735917 | chr05 | SLC6A3 | GoldenGate |
| rs27074 | chr05 |  | GoldenGate |
| rs466630 | chr05 | SLC6A3 | GoldenGate |
| rs246993 | chr05 |  | GoldenGate |
| rs9499708 | chr06 |  | GoldenGate |
| rs1718101 | chr07 | CNTNAP2 | GoldenGate |
| rs769111 | chr07 |  | GoldenGate |
| rs12342908 | chr09 | SLC1A1 | GoldenGate |
| rs7022369 | chr09 | SLC1A1 | GoldenGate |
| rs301979 | chr09 | SLC1A1 | GoldenGate |
| rs12378107 | chr09 | SLC1A1 | GoldenGate |
| rs2039290 | chr09 | SLC1A1 | GoldenGate |
| rs10739064 | chr09 | SLC1A1 | GoldenGate |
| rs10814998 | chr09 | SLC1A1 | GoldenGate |
| rs12341219 | chr09 | SLC1A1 | GoldenGate |
| rs10491735 | chr09 |  | GoldenGate |
| rs10739066 | chr09 | SLC1A1 | GoldenGate |
| rs4742003 | chr09 | SLC1A1 | GoldenGate |
| rs10491731 | chr09 | SLC1A1 | GoldenGate |
| rs7858819 | chr09 | SLC1A1 | GoldenGate |
| rs10974591 | chr09 | SLC1A1 | GoldenGate |
| rs10814991 | chr09 | SLC1A1 | GoldenGate |
| rs12553697 | chr09 |  | GoldenGate |
| rs6476873 | chr09 | SLC1A1 | GoldenGate |
| rs7031998 | chr09 | SLC1A1 | GoldenGate |
| rs10739062 | chr09 | SLC1A1 | GoldenGate |
| rs928209 | chr09 | SLC1A1 | GoldenGate |
| rs301443 | chr09 |  | GoldenGate |
| rs10974616 | chr09 | SLC1A1 | GoldenGate |
| rs9775228 | chr09 |  | GoldenGate |
| rs10758632 | chr09 | SLC1A1 | GoldenGate |
| rs10814993 | chr09 | SLC1A1 | GoldenGate |
| rs301434 | chr09 | SLC1A1 | GoldenGate |
| rs301439 | chr09 |  | GoldenGate |
| rs17812372 | chr09 |  | GoldenGate |
| rs972519 | chr09 | SLC1A1 | GoldenGate |
| rs10814988 | chr09 |  | GoldenGate |
| rs10974620 | chr09 | SLC1A1 | GoldenGate |
| rs301445 | chr09 |  | GoldenGate |
| rs10815013 | chr09 | SLC1A1 | GoldenGate |
| rs10815019 | chr09 | SLC1A1 | GoldenGate |
| rs2587548 | chr11 | DRD2 | GoldenGate |
| rs2587550 | chr11 |  | GoldenGate |
| rs7122454 | chr11 | DRD2 | GoldenGate |
| rs17115583 | chr11 | DRD2 | GoldenGate |
| rs4245147 | chr11 | DRD2 | GoldenGate |
| rs2234689 | chr11 |  | GoldenGate |
| rs1800497 | chr11 | ANKK1 | GoldenGate |
| rs11214606 | chr11 | DRD2 | GoldenGate |
| rs6279 | chr11 | DRD2 | GoldenGate |
| rs5016282 | chr11 | GRM5 | GoldenGate |
| rs4271390 | chr11 | PVRL1 | GoldenGate |
| rs7955501 | chr12 | TPH2 | GoldenGate |
| rs1386497 | chr12 | TPH2 | GoldenGate |
| rs17110536 | chr12 | TPH2 | GoldenGate |
| rs10879358 | chr12 | TPH2 | GoldenGate |
| rs10748185 | chr12 | TPH2 | GoldenGate |
| rs17722134 | chr12 | TPH2 | GoldenGate |
| rs4760813 | chr12 |  | GoldenGate |
| rs17110747 | chr12 | TPH2 | GoldenGate |
| rs11615016 | chr12 | TPH2 | GoldenGate |
| rs12424836 | chr12 |  | GoldenGate |
| rs11149058 | chr13 |  | GoldenGate |
| rs9531520 | chr13 |  | GoldenGate |
| rs8029462 | chr15 | SLC27A2 | GoldenGate |
| rs11856059 | chr15 |  | GoldenGate |
| rs1365503 | chr15 |  | GoldenGate |
| rs854157 | chr15 |  | GoldenGate |
| rs2853766 | chr15 |  | GoldenGate |
| rs854158 | chr15 |  | GoldenGate |
| rs854159 | chr15 |  | GoldenGate |
| rs2070595 | chr15 |  | GoldenGate |
| rs8034597 | chr15 |  | GoldenGate |
| rs854150 | chr15 |  | GoldenGate |
| rs9920021 | chr15 |  | GoldenGate |
| rs662669 | chr17 | TBCD | GoldenGate |
| rs3744161 | chr17 | TBCD | GoldenGate |
| rs3787430 | chr20 | HRH3 | GoldenGate |
| rs1760042 | chr20 |  | GoldenGate |
| rs6062144 | chr20 |  | GoldenGate |
| rs6061458 | chr20 |  | GoldenGate |
| rs3787429 | chr20 | HRH3 | GoldenGate |
| rs6142998 | chr20 |  | GoldenGate |
| rs518147 | chrX | HTR2C | GoldenGate |
| rs5906729 | chrX | MAO-A | GoldenGate |
| rs5905859 | chrX | MAO-A | GoldenGate |
| rs3027399 | chrX | MAO-A | GoldenGate |
| rs12843533 | chrX | MAO-A | GoldenGate |

^a^GoldenGate: Illumina GoldenGate Genotyping Assay;  OmniExpressExome: Illumina HumanOmniExpressExome v1.2 BeadChip array.

Table S2: Investigated SNPs after quality control

| SNP | Gene | Category | Reference |
| --- | --- | --- | --- |
| rs769111 |  | Top SNP GWAS TS | (5) |
| rs7868992 | *COL27A1* | Top SNP GWAS TS | (5) |
| rs11603305 |  | Top SNP GWAS TS | (6) |
| rs621942 | *PICALM* | Top SNP GWAS TS | (6) |
| rs7123010 | *ME3* | Top SNP GWAS TS | (6) |
| rs6539267 | *POLR3B* | Top SNP GWAS TS | (5) |
| rs7336083 |  | Top SNP GWAS TS | (5) |
| rs4675502 | *PARD3B* | Top SNP GWAS OCD/ADHD/ASD | (1) |
| rs4988462 | *POU1F1* | Top SNP GWAS OCD/ADHD/ASD | (2) |
| rs13176113 |  | Top SNP GWAS OCD/ADHD/ASD | (3) |
| rs4307059 |  | Top SNP GWAS OCD/ADHD/ASD | (3) |
| rs7711337 |  | Top SNP GWAS OCD/ADHD/ASD | (1) |
| rs9499708 |  | Top SNP GWAS OCD/ADHD/ASD | (4) |
| rs11081062 | *DLGAP1* | Top SNP GWAS OCD/ADHD/ASD | (4) |
| rs6131295 |  | Top SNP GWAS OCD/ADHD/ASD | (4) |
| rs6347 | *DAT1* | Previously implicated in candidate gene studies of TS | (8) |
| rs9357271 | *BTBD9* | Previously implicated in candidate gene studies of TS | (9) |
| rs6279 | *DRD2* | Previously implicated in candidate gene studies of TS | (11) |
| rs4648318 | *DRD2* | Previously implicated in candidate gene studies of TS | (11) |
| rs4570625 | *TPH2* | Previously implicated in candidate gene studies of TS | (12) |
| rs4565946 | *TPH2* | Previously implicated in candidate gene studies of TS | (12) |
| rs9593835 | *SLITRK1* | Previously implicated in candidate gene studies of TS | (13) |
| rs854150 | *HDC* | Previously implicated in candidate gene studies of TS | (14) |
| rs662669 | *TBCD* | Previously implicated in candidate gene studies of TS | (15) |
| rs3744161 | *TBCD* | Previously implicated in candidate gene studies of TS | (15) |
| rs11264126 | *DLGAP3* | Previously implicated in OCD/ASD | (7) |
| rs7794745 | *CNTNAP2* | Previously implicated in OCD/ASD | (10) |
| rs4680 | *COMT* | Previously implicated in OCD/ASD | (16) |
| rs16859227 | *GABRA2* | TS neurotransmitter-related candidate genes | (17) |
| rs279828 | *GABRA2* | TS neurotransmitter-related candidate genes | (17) |
| rs1442060 | *GABRA2* | TS neurotransmitter-related candidate genes | (17) |
| rs16859354 | *GABRA2* | TS neurotransmitter-related candidate genes | (17) |
| rs10013922 | *GABRA2* | TS neurotransmitter-related candidate genes | (17) |
| rs11503014 | *GABRA2* | TS neurotransmitter-related candidate genes | (17) |
| rs1372472 | *GABRA2* | TS neurotransmitter-related candidate genes | (17) |
| rs1042098 | *DAT1* | TS neurotransmitter-related candidate genes | (8) |
| rs40184 | *DAT1* | TS neurotransmitter-related candidate genes | (8) |
| rs27048 | *DAT1* | TS neurotransmitter-related candidate genes | (8) |
| rs11564758 | *DAT1* | TS neurotransmitter-related candidate genes | (8) |
| rs10052016 | *DAT1* | TS neurotransmitter-related candidate genes | (8) |
| rs2550948 | *DAT1* | TS neurotransmitter-related candidate genes | (8) |
| rs10491734 | *SLC1A1* | TS neurotransmitter-related candidate genes | (18) |
| rs9775228 | *SLC1A1* | TS neurotransmitter-related candidate genes | (18) |
| rs2150192 | *SLC1A1* | TS neurotransmitter-related candidate genes | (18) |
| rs2183472 | *SLC1A1* | TS neurotransmitter-related candidate genes | (18) |
| rs7031998 | *SLC1A1* | TS neurotransmitter-related candidate genes | (18) |
| rs10814991 | *SLC1A1* | TS neurotransmitter-related candidate genes | (18) |
| rs10814993 | *SLC1A1* | TS neurotransmitter-related candidate genes | (18) |
| rs7021409 | *SLC1A1* | TS neurotransmitter-related candidate genes | (18) |
| rs10739062 | *SLC1A1* | TS neurotransmitter-related candidate genes | (18) |
| rs2150195 | *SLC1A1* | TS neurotransmitter-related candidate genes | (18) |
| rs12342908 | *SLC1A1* | TS neurotransmitter-related candidate genes | (18) |
| rs10814998 | *SLC1A1* | TS neurotransmitter-related candidate genes | (18) |
| rs4742003 | *SLC1A1* | TS neurotransmitter-related candidate genes | (18) |
| rs4742004 | *SLC1A1* | TS neurotransmitter-related candidate genes | (18) |
| rs7022369 | *SLC1A1* | TS neurotransmitter-related candidate genes | (18) |
| rs7848533 | *SLC1A1* | TS neurotransmitter-related candidate genes | (18) |
| rs928209 | *SLC1A1* | TS neurotransmitter-related candidate genes | (18) |
| rs10758631 | *SLC1A1* | TS neurotransmitter-related candidate genes | (18) |
| rs10815019 | *SLC1A1* | TS neurotransmitter-related candidate genes | (18) |
| rs12341219 | *SLC1A1* | TS neurotransmitter-related candidate genes | (18) |
| rs10739064 | *SLC1A1* | TS neurotransmitter-related candidate genes | (18) |
| rs10758632 | *SLC1A1* | TS neurotransmitter-related candidate genes | (18) |
| rs10739066 | *SLC1A1* | TS neurotransmitter-related candidate genes | (18) |
| rs10974619 | *SLC1A1* | TS neurotransmitter-related candidate genes | (18) |
| rs7858819 | *SLC1A1* | TS neurotransmitter-related candidate genes | (18) |
| rs7022772 | *SLC1A1* | TS neurotransmitter-related candidate genes | (18) |
| rs7864496 | *SLC1A1* | TS neurotransmitter-related candidate genes | (18) |
| rs301979 | *SLC1A1* | TS neurotransmitter-related candidate genes | (18) |
| rs6476879 | *SLC1A1* | TS neurotransmitter-related candidate genes | (18) |
| rs12378107 | *SLC1A1* | TS neurotransmitter-related candidate genes | (18) |
| rs301434 | *SLC1A1* | TS neurotransmitter-related candidate genes | (18) |
| rs301439 | *SLC1A1* | TS neurotransmitter-related candidate genes | (18) |
| rs301443 | *SLC1A1* | TS neurotransmitter-related candidate genes | (18) |
| rs301445 | *SLC1A1* | TS neurotransmitter-related candidate genes | (18) |
| rs2587550 | *DRD2* | TS neurotransmitter-related candidate genes | (19) |
| rs2587548 | *DRD2* | TS neurotransmitter-related candidate genes | (19) |
| rs17529477 | *DRD2* | TS neurotransmitter-related candidate genes | (19) |
| rs4245147 | *DRD2* | TS neurotransmitter-related candidate genes | (19) |
| rs7131056 | *DRD2* | TS neurotransmitter-related candidate genes | (19) |
| rs11178999 | *TPH2* | TS neurotransmitter-related candidate genes | (20) |
| rs10748185 | *TPH2* | TS neurotransmitter-related candidate genes | (20) |
| rs7955501 | *TPH2* | TS neurotransmitter-related candidate genes | (20) |
| rs17110536 | *TPH2* | TS neurotransmitter-related candidate genes | (20) |
| rs4760820 | *TPH2* | TS neurotransmitter-related candidate genes | (20) |
| rs1487275 | *TPH2* | TS neurotransmitter-related candidate genes | (20) |
| rs10879357 | *TPH2* | TS neurotransmitter-related candidate genes | (20) |
| rs10879358 | *TPH2* | TS neurotransmitter-related candidate genes | (20) |
| rs1872824 | *TPH2* | TS neurotransmitter-related candidate genes | (20) |
| rs2070595 | *HDC* | TS neurotransmitter-related candidate genes | (21) |
| rs2853766 | *HDC* | TS neurotransmitter-related candidate genes | (21) |
| rs854157 | *HDC* | TS neurotransmitter-related candidate genes | (21) |
| rs854158 | *HDC* | TS neurotransmitter-related candidate genes | (21) |
| rs854159 | *HDC* | TS neurotransmitter-related candidate genes | (21) |
| rs9920021 | *HDC* | TS neurotransmitter-related candidate genes | (21) |
| rs1365503 | *HDC* | TS neurotransmitter-related candidate genes | (21) |
| rs6061458 | *HRH3* | TS neurotransmitter-related candidate genes | (22) |
| rs6142998 | *HRH3* | TS neurotransmitter-related candidate genes | (22) |

SNP, singe nucleotide polymorphism; GWAS, genome-wide association study. 

Table S3: Regression models main-effect analyses with and without relatedness as a random effect

|  | Relatedness as random effect |  | Without relatedness as random effect |  |
| --- | --- | --- | --- | --- |
| SNP | **χ** | ***P*** | **F** | ***P*** |
| rs7123010 | 7.95 | 0.0004 | 7.99 | 0.0004 |
| rs11264126 | 4.46 | 0.0120 | 4.22 | 0.0152 |
| rs6061458 | 0.43 | 0.6509 | 4.22 | 0.0152 |
| rs17529477 | 4.55 | 0.0110 | 4.10 | 0.0171 |
| rs7021409 | 2.92 | 0.0552 | 3.19 | 0.0421 |
| rs928209 | 2.88 | 0.0574 | 3.00 | 0.0511 |
| rs9920021 | 3.22 | 0.0411 | 2.82 | 0.0607 |
| rs854158 | 2.38 | 0.0943 | 2.53 | 0.0813 |
| rs301979 | 2.33 | 0.0988 | 2.30 | 0.1017 |
| rs279828 | 2.20 | 0.1127 | 2.26 | 0.1057 |
| rs301439 | 2.46 | 0.0864 | 2.21 | 0.1105 |
| rs12341219 | 2.05 | 0.1299 | 2.20 | 0.1122 |
| rs3744161 | 2.14 | 0.1188 | 2.11 | 0.1230 |
| rs2587550 | 2.23 | 0.1087 | 2.04 | 0.1318 |
| rs1372472 | 2.12 | 0.1215 | 2.02 | 0.1344 |
| rs6476879 | 1.96 | 0.1424 | 2.00 | 0.1364 |
| rs7848533 | 1.77 | 0.1707 | 1.97 | 0.1403 |
| rs1442060 | 1.85 | 0.1582 | 1.73 | 0.1786 |
| rs6279 | 1.92 | 0.1484 | 1.72 | 0.1808 |
| rs7031998 | 1.65 | 0.1935 | 1.70 | 0.1832 |
| rs11081062 | 1.72 | 0.1800 | 1.70 | 0.1840 |
| rs4675502 | 1.58 | 0.2081 | 1.64 | 0.1961 |
| rs6142998 | 0.33 | 0.7201 | 1.64 | 0.1961 |
| rs7022772 | 1.68 | 0.1880 | 1.59 | 0.2045 |
| rs4565946 | 1.66 | 0.1912 | 1.53 | 0.2179 |
| rs16859227 | 1.47 | 0.2317 | 1.48 | 0.2292 |
| rs11503014 | 1.50 | 0.2255 | 1.46 | 0.2332 |
| rs7336083 | 1.36 | 0.2585 | 1.45 | 0.2364 |
| rs11564758 | 1.32 | 0.2685 | 1.39 | 0.2512 |
| rs4742003 | 1.32 | 0.2683 | 1.38 | 0.2532 |
| rs4742004 | 1.22 | 0.2955 | 1.36 | 0.2567 |
| rs10739064 | 1.20 | 0.3022 | 1.29 | 0.2757 |
| rs2587548 | 1.16 | 0.3140 | 1.29 | 0.2773 |
| rs7794745 | 1.37 | 0.2554 | 1.26 | 0.2852 |
| rs7955501 | 1.17 | 0.3119 | 1.20 | 0.3012 |
| rs10491734 | 1.29 | 0.2763 | 1.20 | 0.3023 |
| rs10758632 | 1.14 | 0.3221 | 1.17 | 0.3103 |
| rs7022369 | 1.24 | 0.2903 | 1.16 | 0.3131 |
| rs12342908 | 1.10 | 0.3340 | 1.16 | 0.3137 |
| rs10748185 | 1.17 | 0.3112 | 1.10 | 0.3339 |
| rs13176113 | 1.09 | 0.3388 | 1.06 | 0.3472 |
| rs1487275 | 1.07 | 0.3424 | 1.06 | 0.3477 |
| rs10013922 | 0.95 | 0.3874 | 1.02 | 0.3618 |
| rs2183472 | 1.00 | 0.3696 | 1.01 | 0.3641 |
| rs17110536 | 1.07 | 0.3429 | 1.01 | 0.3646 |
| rs4307059 | 1.05 | 0.3517 | 1.01 | 0.3651 |
| rs10974619 | 0.94 | 0.3925 | 0.99 | 0.3724 |
| rs9775228 | 1.01 | 0.3645 | 0.96 | 0.3847 |
| rs27048 | 0.85 | 0.4275 | 0.96 | 0.3850 |
| rs662669 | 0.96 | 0.3830 | 0.96 | 0.3852 |
| rs10879357 | 0.90 | 0.4089 | 0.85 | 0.4280 |
| rs301443 | 0.94 | 0.3899 | 0.78 | 0.4577 |
| rs7868992 | 0.67 | 0.5139 | 0.76 | 0.4666 |
| rs2150195 | 0.91 | 0.4040 | 0.75 | 0.4711 |
| rs1042098 | 0.66 | 0.5152 | 0.70 | 0.4953 |
| rs9499708 | 0.65 | 0.5217 | 0.69 | 0.4999 |
| rs9593835 | 0.71 | 0.4944 | 0.68 | 0.5088 |
| rs4680 | 0.36 | 0.6971 | 0.64 | 0.5269 |
| rs4988462 | 0.69 | 0.5032 | 0.64 | 0.5269 |
| rs854150 | 0.67 | 0.5147 | 0.61 | 0.5443 |
| rs7858819 | 0.58 | 0.5625 | 0.59 | 0.5549 |
| rs6539267 | 0.55 | 0.5784 | 0.55 | 0.5780 |
| rs10052016 | 0.56 | 0.5725 | 0.53 | 0.5881 |
| rs10814998 | 0.48 | 0.6193 | 0.53 | 0.5890 |
| rs621942 | 0.52 | 0.5937 | 0.52 | 0.5944 |
| rs2550948 | 0.52 | 0.5972 | 0.49 | 0.6136 |
| rs10758631 | 0.36 | 0.6969 | 0.47 | 0.6274 |
| rs16859354 | 0.42 | 0.6580 | 0.47 | 0.6284 |
| rs7131056 | 0.48 | 0.6206 | 0.45 | 0.6384 |
| rs40184 | 0.40 | 0.6688 | 0.44 | 0.6457 |
| rs10815019 | 0.45 | 0.6362 | 0.43 | 0.6480 |
| rs4570625 | 0.37 | 0.6929 | 0.41 | 0.6666 |
| rs11178999 | 0.40 | 0.6694 | 0.40 | 0.6720 |
| rs4245147 | 0.39 | 0.6780 | 0.34 | 0.7142 |
| rs1872824 | 0.28 | 0.7565 | 0.33 | 0.7174 |
| rs10814993 | 0.34 | 0.7129 | 0.32 | 0.7256 |
| rs7864496 | 0.36 | 0.6953 | 0.29 | 0.7475 |
| rs1365503 | 0.36 | 0.6958 | 0.29 | 0.7491 |
| rs6347 | 0.25 | 0.7804 | 0.29 | 0.7498 |
| rs301434 | 0.39 | 0.6768 | 0.28 | 0.7551 |
| rs10739062 | 0.38 | 0.6856 | 0.27 | 0.7613 |
| rs854159 | 0.19 | 0.8265 | 0.24 | 0.7897 |
| rs2150192 | 0.28 | 0.7560 | 0.23 | 0.7945 |
| rs10814991 | 0.29 | 0.7456 | 0.23 | 0.7983 |
| rs9357271 | 0.25 | 0.7754 | 0.23 | 0.7986 |
| rs2853766 | 0.26 | 0.7748 | 0.22 | 0.8002 |
| rs2070595 | 0.24 | 0.7875 | 0.21 | 0.8072 |
| rs301445 | 0.24 | 0.7831 | 0.21 | 0.8116 |
| rs854157 | 0.15 | 0.8640 | 0.19 | 0.8283 |
| rs10879358 | 0.21 | 0.8125 | 0.19 | 0.8305 |
| rs7711337 | 0.13 | 0.8766 | 0.18 | 0.8329 |
| rs10739066 | 0.26 | 0.7725 | 0.17 | 0.8415 |
| rs4760820 | 0.12 | 0.8907 | 0.16 | 0.8562 |
| rs769111 | 0.07 | 0.9337 | 0.07 | 0.9340 |
| rs12378107 | 0.05 | 0.9486 | 0.06 | 0.9406 |
| rs6131295 | 0.11 | 0.8994 | 0.05 | 0.9470 |
| rs4648318 | 0.08 | 0.9207 | 0.03 | 0.9720 |
| rs11603305 | 0.01 | 0.9869 | 0.01 | 0.9922 |

SNP, singe nucleotide polymorphism

Table S4: Regression models interaction analyses with and without relatedness as a random effect

|  | Relatedness as random effect |  | Relatedness as random effect |  |
| --- | --- | --- | --- | --- |
| SNP | **χ** | ***P*** | **F** | ***P*** |
| rs6539267 | 6.94 | 0.001 | 6.80 | 0.001 |
| rs12342908 | 4.81 | 0.009 | 4.70 | 0.010 |
| rs4742003 | 4.15 | 0.017 | 4.05 | 0.018 |
| rs4648318 | 3.65 | 0.027 | 3.58 | 0.029 |
| rs301979 | 3.60 | 0.028 | 3.51 | 0.031 |
| rs2150195 | 3.29 | 0.038 | 3.23 | 0.040 |
| rs4742004 | 3.19 | 0.042 | 3.13 | 0.045 |
| rs2587550 | 2.69 | 0.069 | 2.63 | 0.074 |
| rs279828 | 2.68 | 0.070 | 2.62 | 0.074 |
| rs6279 | 2.59 | 0.076 | 2.53 | 0.081 |
| rs10758632 | 2.48 | 0.085 | 2.42 | 0.090 |
| rs7858819 | 2.32 | 0.100 | 2.27 | 0.105 |
| rs10815019 | 2.04 | 0.131 | 2.00 | 0.137 |
| rs854158 | 1.99 | 0.139 | 1.94 | 0.145 |
| rs10814998 | 1.94 | 0.145 | 1.89 | 0.152 |
| rs10814991 | 1.93 | 0.147 | 1.89 | 0.153 |
| rs10013922 | 1.92 | 0.148 | 1.88 | 0.154 |
| rs10814993 | 1.79 | 0.168 | 1.75 | 0.175 |
| rs10739064 | 1.77 | 0.172 | 1.73 | 0.179 |
| rs7864496 | 1.75 | 0.175 | 1.72 | 0.181 |
| rs17529477 | 1.66 | 0.191 | 1.63 | 0.198 |
| rs9775228 | 1.63 | 0.198 | 1.59 | 0.205 |
| rs27048 | 1.52 | 0.221 | 1.49 | 0.227 |
| rs40184 | 1.51 | 0.223 | 1.48 | 0.229 |
| rs854159 | 1.50 | 0.224 | 1.47 | 0.231 |
| rs2853766 | 1.48 | 0.228 | 1.46 | 0.234 |
| rs4988462 | 1.47 | 0.231 | 1.44 | 0.239 |
| rs4680 | 0.21 | 0.809 | 1.44 | 0.239 |
| rs2183472 | 1.25 | 0.288 | 1.22 | 0.295 |
| rs854157 | 1.23 | 0.294 | 1.20 | 0.302 |
| rs11264126 | 1.16 | 0.315 | 1.14 | 0.322 |
| rs6061458 | 2.54 | 0.080 | 1.14 | 0.322 |
| rs10739062 | 1.11 | 0.331 | 1.08 | 0.339 |
| rs2587548 | 1.07 | 0.345 | 1.04 | 0.353 |
| rs12341219 | 1.00 | 0.368 | 0.98 | 0.376 |
| rs7336083 | 1.00 | 0.370 | 0.98 | 0.377 |
| rs6347 | 0.98 | 0.376 | 0.96 | 0.385 |
| rs301434 | 0.91 | 0.402 | 0.89 | 0.410 |
| rs11503014 | 0.90 | 0.408 | 0.88 | 0.416 |
| rs7711337 | 0.87 | 0.419 | 0.86 | 0.426 |
| rs9920021 | 0.87 | 0.418 | 0.85 | 0.426 |
| rs10974619 | 0.87 | 0.421 | 0.85 | 0.428 |
| rs1042098 | 0.85 | 0.426 | 0.84 | 0.433 |
| rs928209 | 0.80 | 0.449 | 0.78 | 0.457 |
| rs7131056 | 0.78 | 0.461 | 0.76 | 0.468 |
| rs6476879 | 0.75 | 0.472 | 0.74 | 0.479 |
| rs16859354 | 0.74 | 0.479 | 0.72 | 0.487 |
| rs16859227 | 0.73 | 0.481 | 0.72 | 0.488 |
| rs10879357 | 0.71 | 0.494 | 0.69 | 0.501 |
| rs1442060 | 0.70 | 0.496 | 0.69 | 0.503 |
| rs301439 | 0.70 | 0.496 | 0.69 | 0.504 |
| rs10491734 | 0.69 | 0.503 | 0.67 | 0.510 |
| rs7955501 | 0.67 | 0.512 | 0.66 | 0.520 |
| rs11178999 | 0.66 | 0.517 | 0.65 | 0.523 |
| rs301443 | 0.65 | 0.525 | 0.63 | 0.532 |
| rs4570625 | 0.63 | 0.535 | 0.61 | 0.542 |
| rs4565946 | 0.62 | 0.537 | 0.61 | 0.544 |
| rs11603305 | 0.63 | 0.534 | 0.61 | 0.546 |
| rs10739066 | 0.61 | 0.543 | 0.60 | 0.550 |
| rs9499708 | 0.60 | 0.547 | 0.59 | 0.554 |
| rs7021409 | 0.57 | 0.564 | 0.56 | 0.571 |
| rs7848533 | 0.57 | 0.565 | 0.56 | 0.571 |
| rs4245147 | 0.54 | 0.583 | 0.53 | 0.590 |
| rs10748185 | 0.54 | 0.584 | 0.53 | 0.591 |
| rs301445 | 0.53 | 0.592 | 0.51 | 0.599 |
| rs10879358 | 0.52 | 0.592 | 0.51 | 0.599 |
| rs17110536 | 0.50 | 0.610 | 0.48 | 0.616 |
| rs10758631 | 0.49 | 0.611 | 0.48 | 0.616 |
| rs7022772 | 0.46 | 0.634 | 0.45 | 0.639 |
| rs4307059 | 0.45 | 0.635 | 0.44 | 0.642 |
| rs4675502 | 0.44 | 0.647 | 0.43 | 0.653 |
| rs6142998 | 0.22 | 0.803 | 0.43 | 0.653 |
| rs1372472 | 0.38 | 0.686 | 0.37 | 0.692 |
| rs12378107 | 0.37 | 0.690 | 0.36 | 0.696 |
| rs7868992 | 0.35 | 0.704 | 0.34 | 0.708 |
| rs9357271 | 0.33 | 0.721 | 0.32 | 0.726 |
| rs621942 | 0.30 | 0.742 | 0.29 | 0.750 |
| rs4760820 | 0.28 | 0.753 | 0.28 | 0.757 |
| rs9593835 | 0.28 | 0.753 | 0.28 | 0.757 |
| rs3744161 | 0.26 | 0.769 | 0.26 | 0.773 |
| rs11564758 | 0.26 | 0.772 | 0.25 | 0.777 |
| rs1487275 | 0.25 | 0.778 | 0.25 | 0.782 |
| rs1872824 | 0.24 | 0.783 | 0.24 | 0.788 |
| rs7794745 | 0.23 | 0.796 | 0.22 | 0.799 |
| rs6131295 | 0.22 | 0.799 | 0.22 | 0.802 |
| rs7022369 | 0.22 | 0.807 | 0.21 | 0.810 |
| rs2070595 | 0.21 | 0.809 | 0.21 | 0.812 |
| rs1365503 | 0.19 | 0.825 | 0.19 | 0.828 |
| rs11081062 | 0.12 | 0.884 | 0.12 | 0.886 |
| rs769111 | 0.11 | 0.899 | 0.10 | 0.901 |
| rs10052016 | 0.09 | 0.913 | 0.09 | 0.915 |
| rs2550948 | 0.07 | 0.930 | 0.07 | 0.932 |
| rs7031998 | 0.05 | 0.951 | 0.05 | 0.952 |
| rs13176113 | 0.04 | 0.956 | 0.04 | 0.957 |
| rs662669 | 0.02 | 0.977 | 0.02 | 0.977 |
| rs7123010 | 0.02 | 0.980 | 0.02 | 0.980 |
| rs854150 | 0.01 | 0.986 | 0.01 | 0.986 |
| rs2150192 | 0.01 | 0.993 | 0.01 | 0.993 |

SNP, singe nucleotide polymorphism
